# Quality of Life, Physical Activity Participation, and Perceptions of Physical Rehabilitation Among Community-Reintegrated Veterans With Lower Limb Amputation in Sri Lanka: Convergent Parallel Mixed Methods Study

**DOI:** 10.2196/52811

**Published:** 2024-06-13

**Authors:** Ashan Wijekoon, Dilanthi Gamage Dona, Subashini Jayawardana, Abigail Beane

**Affiliations:** 1 National Intensive Care Surveillance Mahidol Oxford Tropical Medicine Research Unit Colombo Sri Lanka; 2 Department of Allied Health Sciences Faculty of Medicine University of Colombo Colombo Sri Lanka; 3 Department of Health and Care Professions Faculty of Health and Wellbeing University of Winchester Winchester United Kingdom; 4 Mahidol-Oxford Tropical Medicine Research Unit Faculty of Tropical Medicine Mahidol University Bangkok Thailand; 5 Centre for Inflammation Research University of Edinburgh Scotland United Kingdom

**Keywords:** amputation, community-based rehabilitation, lower limb, military, physical activity, quality of life

## Abstract

**Background:**

Lower limb amputation (LLA) impacts physical activity (PA) participation and quality of life (QoL). To minimize the effects of these challenges, LLA survivors need to have opportunities to engage in appropriately tailored rehabilitation throughout their lives. However, in Sri Lanka, where a 3-decade civil war resulted in trauma-related LLA among young male soldiers, access to rehabilitation was limited to the immediate postinjury period. Developing rehabilitation interventions for these veterans requires an understanding of their current health status and rehabilitation perceptions.

**Objective:**

This study was conducted to evaluate the QoL and PA participation of veterans with LLA and explore perceptions of factors influencing their PA participation and expectations for a future community-based physical rehabilitation (CBPR) intervention.

**Methods:**

This mixed methods study combined a comparative cross-sectional quantitative survey with qualitative semistructured interviews in 5 districts of Sri Lanka. QoL and PA participation were assessed among community-reintegrated veterans with LLA (n=85) and compared with a matched able-bodied cohort (control; n=85) using Mann-Whitney *U* and Chi-square tests. PA was assessed in terms of metabolic equivalent of task (MET) minutes per week and was computed for walking, moderate-intensity, and vigorous-intensity activities. PA was classified as sufficiently active, low, or sedentary. The design of interview questions was guided by the Theoretical Domains Framework and followed a phenomenological approach. Interviews were conducted with 25 veterans and were analyzed thematically, and the perceptions regarding PA participation and CBPR were codified using the Consolidated Framework for Implementation Research (CFIR).

**Results:**

Based on the quantitative survey findings, scores for both physical (*P*<.001) and psychological (*P*<.001) well-being and participation in walking (*P*=.004) and vigorous-intensity activities (*P*<.001) were significantly lower among veterans than among controls. A “sedentary” classification was made for 43% (34/79) of veterans and 12% (10/82) of controls. Veterans mostly engaged in moderate-intensity PA inside the house (49/79, 62%) and in the yard (30/79, 38%). Qualitative interviews revealed that barriers to PA exist at individual (eg, comorbidity burden), primary care (eg, absence of community rehabilitation services), and policy levels (eg, limited resources) and facilitators exist primarily at societal (eg, inclusive community) and individual levels (eg, preinjury activity baseline and positive attitudes toward exercise). Expectations regarding CBPR included individualized rehabilitation parameters; functional exercises; and involvement of peers, amputee societies, and community health care providers. The nonresponse rate for interviews was 7% (2/27).

**Conclusions:**

The findings of reduced PA participation, poor QoL, and physical and psychological impairments among relatively young veterans reveal the long-term impacts of living with LLA in the absence of long-term rehabilitation. Policy-level changes need to be implemented along with behavior-change strategies to promote PA participation and minimize physical inactivity–induced health issues. Veterans’ perceptions regarding future CBPR programs were positive and centered on holistic, individualized, and peer-led activities.

## Introduction

Lower limb amputation (LLA) accounts for over 90% of all amputations [1] and is associated with significant morbidity, mortality, and disability [2]. Chronic conditions associated with increased prevalence of cardiovascular diseases and poor long-term quality of life (QoL) outcomes in the LLA population [3-7] are thought to be in part (as a consequence of reduced mobility and chronic pain) compounded by lifestyle choices (diet, alcohol consumption, and smoking) and limited employment opportunities, leading to lower income and economic insecurity [8-10].

War-related trauma is a major cause of LLA in the military population. In Sri Lanka, with its relatively recent history of a long civil war, this trauma accounts for the majority of LLA cases [11]. More than 20,000 military veterans are estimated to be living with disabilities in Sri Lanka, and LLA, either with or without additional injuries, is thought to be the most prevalent physical disability. Given the age and demography of serving officers, war-related traumatic LLA occurs at a young age. As a consequence, those who survive the injury face a future of having to adjust to living and working in civilian society with both their primary disability and associated secondary conditions.

The long-term impacts of both the primary physical injury and sequential health and lifestyle-related conditions in LLA can, however, be mitigated by engagement in regular physical activity (PA) [12,13]. PA is defined as any voluntary bodily movement produced by skeletal muscles that results in energy expenditure and is performed during any time of the day or night [14]. According to the American College of Sports Medicine (ACSM) and American Heart Association (AHA) guidelines, adults aged 18 to 65 years are recommended to perform moderate-intensity aerobic PA for a minimum of 30 minutes a day for 5 days a week or vigorous-intensity aerobic activity for a minimum of 20 minutes a day for 3 days a week [15].

There are several factors known to affect PA participation following LLA. These include present health conditions, provision of informal (family) and formal (health care provider) support, availability of and access to rehabilitation resources, prosthetic function, physical fitness, personal attitude, and knowledge or awareness of the condition [9,16-19]. Moreover, engaging in PA as part of a physical rehabilitation program is more beneficial than performing PA alone, as physical rehabilitation programs further seek to improve chronic pain [20] and balance [21], and increase cardiopulmonary endurance [22,23].

Sustained PA is a major determinant of recovering and maintaining QoL in the LLA population [3,24]. Previous studies on the QoL of Sri Lankan military personnel injured during the civil war, which were based on the Short-Form Health Survey-36 (SF-36), suggested that the presence of comorbidities and limited use of prosthetics are associated with lower QoL [5,25]. Given the potential to mitigate comorbidities and enhance prosthesis use through increased PA [12,26,27] and the strong positive correlation between PA and QoL [3,13], the promotion of PA is a promising avenue to enhance QoL among individuals with LLA.

Currently, there is no formal or government-led rehabilitation service to promote or maintain adequate PA participation for community-reintegrated veterans following LLA in Sri Lanka [28]. Therefore, implementing a tailored community-based physical rehabilitation (CBPR) program could improve PA participation and overall QoL among them. However, this requires an understanding of their current QoL and PA levels, and perceptions of PA and rehabilitation are crucial for developing a feasible and acceptable intervention.

The purpose of this mixed methods study was to understand the current health status in terms of QoL and PA participation and the perceptions of rehabilitation among veterans following LLA in Sri Lanka for informing the development of a future CBPR program. The quantitative and qualitative objectives were as follows:

1. Quantitative objective: To assess QoL outcomes and the level of PA among veterans with LLA in Sri Lanka.
2. Qualitative objective: To explore the factors influencing veterans’ PA participation and their perceptions regarding priorities for and implementation of a CBPR program for individuals living with LLA in Sri Lanka.

## Methods

### Ethics Approval

This study was approved by the Ethics Review Committee of the Faculty of Medicine, University of Colombo, Sri Lanka (EC-19-074).

### Study Design

A mixed methods study involving a convergent parallel approach was conducted [29], and quantitative and qualitative data collection and analyses were carried out concurrently and independently. Findings from both sets of data were integrated to inform the development of a future CBPR intervention for the underlying population. We defined CBPR as an exercise-based rehabilitation intervention practiced in the community or at the home of the participant [22].

#### Quantitative Assessment

A descriptive cross-sectional survey with a comparison group was conducted. We included a comparison group as we wanted to compare the outcomes of veterans with those of able-bodied controls (matched to age and sex) living in the same geographical location, having similar socioeconomic and lifestyle contexts, and having access to similar health care resources.

#### Qualitative Assessment

Qualitative semistructured interviews were conducted using a phenomenological approach. This approach was chosen to encourage the identification of broader emerging themes that crosscut the diverse health, social, societal, and individual factors known to affect engagement in and effectiveness of rehabilitation with regard to PA and QoL. Interviews were designed based on methods described by Creswell [30] in planning and conducting qualitative research and published studies focused on factors influencing PA participation among individuals with chronic disabilities, including LLA [16,19,31].

### Study Setting

The study was conducted in the following 5 districts of Sri Lanka (out of 22) identified based on a priori knowledge of the locations of veterans’ community settlements: Anuradhapura, Kurunegala, Hambanthota, Badulla, and Rathnapura. These 5 districts in Sri Lanka have the highest number of LLA veterans, comprising more than 50% according to the “Disabled Category Registry” manually updated by the Directorate of Rehabilitation, Ministry of Defense, Sri Lanka [11]. Veterans were living in “Ranaviru Villages,” which are located far away from the city center of these districts. “Ranaviru Villages” are residences constructed by the Sri Lankan Army for injured and retired Army veterans. The period of the study was from October 2020 to April 2021.

### Participant Recruitment

#### Quantitative Assessment

We identified potential veterans with LLA (group 1) from the “Disabled Category Registry.” We aimed to include 85 participants in each of the groups so as to adequately power the comparison of each outcome [32]. We ensured representation from veterans across all 5 districts, selecting participants proportionally using a stratified random sampling procedure [33,34]. Participants for the comparison group (group 2) were identified from the same village or a neighboring village of their group 1 counterparts using the voter registration list.

We selected veterans who had LLA due to an injury on the battlefield and were living in the community. To ensure that the participants had the required functional level for the proposed CBPR intervention, we included only participants who had unilateral LLA and used a prosthetic limb for walking and standing activities. Veterans older than 70 years and those with comorbidities that interfered with their function beyond that of unilateral LLA (eg, dependence on renal replacement therapy) were excluded.

#### Qualitative Assessment

For the interviews, we purposively selected participants from group 1, ensuring participation from all 5 districts with regard to transfemoral and transtibial amputations to assess the needs and understand the perspectives of individuals with different functional levels of mobility after amputation [35,36].

### Data Collection

#### Quantitative Assessment

The self-administered SF-36 [37] and International Physical Activity Questionnaire (IPAQ) long-form survey [38] were used to assess QoL and PA participation, respectively. The SF-36 is widely used to measure QoL in terms of physical (physical component summary [PCS]) and mental or emotional (mental component summary [MCS]) components, each expressed as a value between 0 and 100, with a high score representing a better QoL [37]. The IPAQ measures the frequency (days per week), duration (minutes), and level of intensity (vigorous, moderate, walking, or sitting) of PA during the last 7 days [38]. Both questionnaires have established psychometric properties making them ideal for use in the LLA population [25,39,40] and have been validated for use in the Sinhalese population previously [41,42].

Initial contact with the participants was made through Grama Niladhari (GN) and officers of societies of amputee veterans (eg, Ranaviru Sansadaya). GN is a Sri Lankan public official appointed by the central government to carry out administrative duties in a GN division (geographic region), which is a subunit of the divisional secretariat.

Participants of groups 1 and 2 were met by 2 research team members (AW and Dasun Isurinda). AW explained the research, provided participants with study information, and sought consent. AW is a trained physiotherapist fluent in Sinhala, with over 6 years of experience working in both clinical and research capacities within community settings in Sri Lanka. Dasun Isurinda is a practicing physiotherapist with more than 5 years of experience in both inpatient and community physiotherapy settings. The SF-36 and IPAQ were available to participants in paper form in the local language (Sinhala). AW and Dasun Isurinda remained with the participants during the survey completion to answer any questions the participants may have regarding the self-assessment.

#### Qualitative Assessment

The interview guide was developed using the Theoretical Domains Framework (TDF) [43]. This included knowledge about PA or exercises and exercise programs, intentions for participating in PA, environmental context and resources, emotions on life with LLA, and reinforcement through support. The guide was translated by a bilingual research team member (AW) and then back-translated and checked for accuracy by a second researcher (SJ). It was piloted with veterans with LLA who were not included in the final analysis. The pilot resulted in the simplification of the question format. The final survey can be found in Multimedia Appendix 1.

A total of 25 interviews were conducted in the language preferred by the participants (Sinhala) and lasted between 30 and 40 minutes. All the interviews were conducted by the author AW at the residence or home of each participant.

### Data Analysis

#### Quantitative Assessment

All the statistical analyses were performed by author DGD (a qualified statistician; independent of participant allocation and data collection) using STATA/IC for Mac v16.1 (StataCorp). The normality of data distributions was tested with the Shapiro-Wilk test, and data are summarized as mean (SD), median (range), or number (percentage), as appropriate. The Mann-Whitney *U* test and chi-square test were used to evaluate comparisons between groups for continuous and nominal variables, with a significance level of .05.

Data from the IPAQ were processed and reported according to the Guidelines for Data Processing and Analysis of the IPAQ [44]. In the IPAQ, PA is defined in terms of the metabolic equivalent of task (MET) minutes per week, and the questionnaire assesses PA participation in walking, moderate-intensity, and vigorous-intensity activities across 4 domains: work, transport, domestic and garden, and leisure. We computed the PA participation of groups 1 and 2 separately for each of these domains and calculated the total PA level by adding them together. Finally, the level of PA was classified as either sedentary (<600 MET-minutes/week), low (600-3000 MET-minutes/week), or sufficiently active (>3000 MET-minutes/week), based on the total MET-minutes/week [44] for both groups.

#### Qualitative Assessment

The findings of the qualitative study were reported using the Consolidated Criteria for Reporting Qualitative Studies guidelines [45]. Findings were thematically analyzed using the Consolidated Framework for Implementation Research (CFIR). The aim of using the CFIR was to identify the different organizational levels to which the identified barriers, facilitators, and expectations for a future CBPR intervention belong, in order to gain further insights into the effective design and implementation of the intervention considering each organizational level. The CFIR is a pragmatic meta-theoretical framework that helps to identify determinants of a health care intervention implementation with consideration for context, the complexity of the intervention, individual characteristics, and organizational or system-level factors that may facilitate or inhibit implementation [46-48].

Thematic analysis was used to identify emerging themes from the interview responses [30]. Responses were initially reviewed independently by 2 researchers (AW and Nilu Dullewe, both qualified health care professionals trained in qualitative methods and fluent in the Sinhala language) who read through all the verbatim transcripts to inductively code sentences and keywords. Emerging themes were then codified using the domains of the CFIR. These were then reviewed by both researchers, duplicates were removed, and emergent themes were refined. Any disagreements that developed during the analysis were discussed, and if needed, these were further reviewed by the author AB, a clinical researcher with experience of both the Sri Lankan health care setting and the methods used for analysis.

### Integration

The themes identified through qualitative analysis were mapped with the findings from quantitative analysis to enhance our understanding of the factors influencing QoL outcomes and PA participation among veterans. Additionally, themes of the barriers and facilitators to PA were transformed into quantitative scores to understand the importance of each theme and its relevance to quantitative analysis findings.

## Results

### Participant Characteristics

In total, 170 individuals (85 in each group) participated in the study, and they represented 5 districts. Table 1 presents the sociodemographic and clinical characteristics of groups 1 and 2.

All the veterans were active prosthetic users who had undergone amputation as a result of battlefield trauma more than 10 years ago. Of the 85 veterans, 78 (92%) had transtibial amputation and 7 (8%) had transfemoral amputation. A high prevalence of amputation-associated comorbidities was found among the veterans. These data have been published separately [49].

All the veterans had completed prosthetic training during postsurgical hospital care. Upon discharge, the veterans were advised to follow a lower limb muscle strengthening and stretching routine thrice a week for 6 months by physiotherapists, but only 12 out of the 85 veterans (14.1%) had engaged as recommended, with an additional 4 veterans (4.7%) following the routine on an ad hoc basis. No participants received follow-up from rehabilitation providers, and none were engaged in health care–administered physical rehabilitation. Moreover, 3 veterans (3.5%) pursued self-directed exercise programs involving social media videos to reduce body weight and manage back pain.

**Table 1:** Sociodemographic and clinical characteristics of the participants.

Characteristic			Group 1 (n=85)	Group 2 (n=85)
Male gender, n (%)			85 (100)	85 (100)
Age (years), mean (SD)			46.3 (6.0)	46.7 (6.0)
BMI (kg/m^2^), mean (SD)			26.2 (3.4)	25.0 (3.1)
War-related traumatic amputation, n (%)			85 (100)	—^a^
Time since amputation (years), mean (SD)			21.7 (5.9)	—
Prosthesis use (hours/day), mean (SD)			14.3 (2.4)	—
Amputation type (unilateral), n (%)			85 (100)	—
**Amputation level, n (%)**				
	Transfemoral	7 (8)		—
	Transtibial	78 (92)		—
**Marital status, n (%)**				
	Single	8 (9)		14 (17)
	Married	71 (84)		66 (78)
	Divorced, separated, or widowed	6 (7)		5 (6)
**Highest education level, n (%)**				
	Grade 6-10	47 (55)		35 (41)
	Passed GCE^b^ Ordinary Level	23 (27)		28 (33)
	Grade 11-13	4 (5)		7 (8)
	Passed GCE Advanced Level	3 (4)		6 (7)
	Vocational training or diploma	7 (8)		5 (6)
	First degree	1 (1)		4 (5)
**Current employment status, n (%)**				
	Employed or self-employed	62 (73)		75 (88)
	Not employed	23 (27)		10 (12)
**Monthly income (LKR^c^), n (%)**				
	<20,000	0 (0)		2 (2)
	20,000-29,999	15 (18)		19 (22)
	30,000-39,000	37 (44)		28 (33)
	≥40,000	33 (39)		36 (42)

^a^Not applicable.

^b^GCE: General Certificate of Education.

^c^A currency exchange rate of 1 LKR=0.0033 USD is applicable.

### Quantitative Assessment

#### QoL Outcomes (SF-36 Scores)

QoL scores by SF-36 domains are presented in Table 2. The median cumulative scores of physical health (PCS) and psychological well-being (MCS) were significantly lower in group 1 than in group 2 (*P*<.001). The difference in the PCS score had a large effect size (*r*=0.5), while the difference in the MCS score had a medium effect size (*r*=0.3). For group 1 participants, the poorest QoL scores were related to general health (median 45, IQR 55-35) (Table 2).

In the comparison of QoL outcomes between different amputation levels, only the “general health” domain (under PCS) showed a significant difference, with a lower value for veterans with transfemoral amputation (*P*=.009; Multimedia Appendix 2).

**Table 2:** Comparison of quality of life outcomes (Short-Form Health Survey-36) between group 1 (veterans with lower limb amputation) and group 2 (able-bodied controls).

Quality of life domain			Group 1 (n=85), median (IQR)		Group 2 (n=85), median (IQR)		*P* value^a^
**Physical health**							
	Physical functioning	60 (72.5-45)		90 (100-80)^b^		<.001	
	Role limitation due to physical problems	50 (75-25)		75 (100-50)^b^		<.001	
	Bodily pain	67.5 (77.5-55)		77.5 (90-67.5)^b^		<.001	
	General health	45 (55-35)		60 (70-50)^b^		<.001	
	Physical health component	54.4 (65.9-44.7)		73.1 (83.4-64.1)^b^		<.001	
**Mental health**							
	Role limitation due to emotional problems	66.7 (100-33.3)		100 (100-33.3)^b^		.01	
	Social functioning	75 (87.5-62.5)		87.5 (87.5-75)^b^		<.001	
	Vitality	60 (70-50)		65 (77.5-57.5)^b^		<.001	
	Emotional well-being	52 (60-48)		56 (60-52)		.40	
	Mental health component	61.8 (71.3-48.9)		72.0 (78.7-60.1)^b^		<.001	

^a^Statistical significance was assessed using the Mann-Whitney *U* test for comparisons between group 1 and group 2.

^b^Statistical significance at *P*<.05.

#### PA Participation (IPAQ Scores)

The total PA level was significantly lower in group 1 than in group 2 (*P*<.001), with a medium effect size (*r*=0.3). Participation in walking, moderate-intensity, and vigorous-intensity activities was lower in group 1 than in group 2, with a significant difference in walking (small effect size of *r*=0.2; *P*=.004) and vigorous-intensity PA (medium effect size of *r*=0.3; *P*<.001) (Table 3). Among 79 veterans, 59 (75%) did not meet the recommended PA level (>3000 MET-minutes/week). Moreover, the “sedentary” level was noted in 43% (34/79) of participants in group 1 and 12% (10/82) of participants in group 2 (*P*<.001) (Table 4).

Of the 79 participants with LLA, the majority engaged in moderate-intensity PA inside the house (49/79, 62%) and in the yard (30/79, 38%). The least participation was in cycling for transport (5/79, 6%) and vigorous PA (recreation, sport, or exercise) in leisure (6/79, 8%) (Multimedia Appendix 3).

When considering the amputation level, participation in walking was significantly lower among veterans with transfemoral amputation than among those with transtibial amputation (*P*=.01), and 4 out of the 5 participants (80%) with transfemoral amputation had PA levels below the recommended guidelines (Multimedia Appendix 2).

**Table 3:** Comparison of physical activity participation between group 1 (veterans with lower limb amputation) and group 2 (able-bodied controls).

Variable			Group 1 (n=79), median (IQR)		Group 2 (n=82), median (IQR)		*P* value^a^
Total physical activity level (MET^b^-minutes/week)			1913.6 (3506.9-515.8)		4857.3 (8296.0-1008.4)^c^		<.001
**Physical activity domain (** **MET-minutes/week)**							
	Work	0.0 (0.0-611.5)		590.6 (0.0-3956.8)^c^		<.001	
	Transport	0.0 (0.0-207.9)		155.9 (0.0-462.6)^c^		.003	
	Domestic and garden	756 (401.6-2236.5)		787.0 (265.8-2457.0)		.79	
	Leisure	0.0 (0.0-140.9)		0.0 (0.0-359.8)		.06	
**Physical activity intensity (** **MET-minutes/week)**							
	Total walking	145.5 (0.0-644.5)		519.6 (64.9-1164.2)^c^		.004	
	Total moderate-intensity activity	1134.0 (476.4-3039.6)		1260.0 (584.8-3169.7)		.21	
	Total vigorous-intensity activity	0.0 (0.0-189.0)		126.0 (0.0-3024.0)^c^		<.001	

^a^Statistical significance was assessed using the Mann-Whitney *U* test for comparisons between group 1 and group 2.

^b^MET: metabolic equivalent of task.

^c^Statistical significance at *P*<.05.

**Table 4:** Comparison of physical activity behaviors between group 1 (veterans with lower limb amputation) and group 2 (able-bodied controls)

		Group 1 (n=79), n (%)		Group 2 (n=82), n (%)		Chi-square (*df*)		*P* value^a^
**Physical activity behavior**						17.66 (2)		<.001
	Sedentary	34 (43)		10 (12)				
	Low	25 (32)		33 (40)				
	Sufficiently active	20 (25)		39 (48)				

^a^Statistical significance was assessed using the Chi-square test for comparison between group 1 and group 2.

### Qualitative Assessment

Of the 79 participants in group 1 who completed the assessment of QoL and PA, 27 (32%) were invited to participate in the semistructured interviews, and of these, 25 consented to participate. Accordingly, 25 interviews were conducted, with a total of 7.2 hours of transcription data. Participants were aged 30 to 55 years (mean 46.1, SD 7.4 years). Moreover, 20 (80%) participants had transtibial LLA and 5 (20%) had transfemoral LLA.

### Barriers and Facilitators to PA Participation in the Community

**Figure 1:**
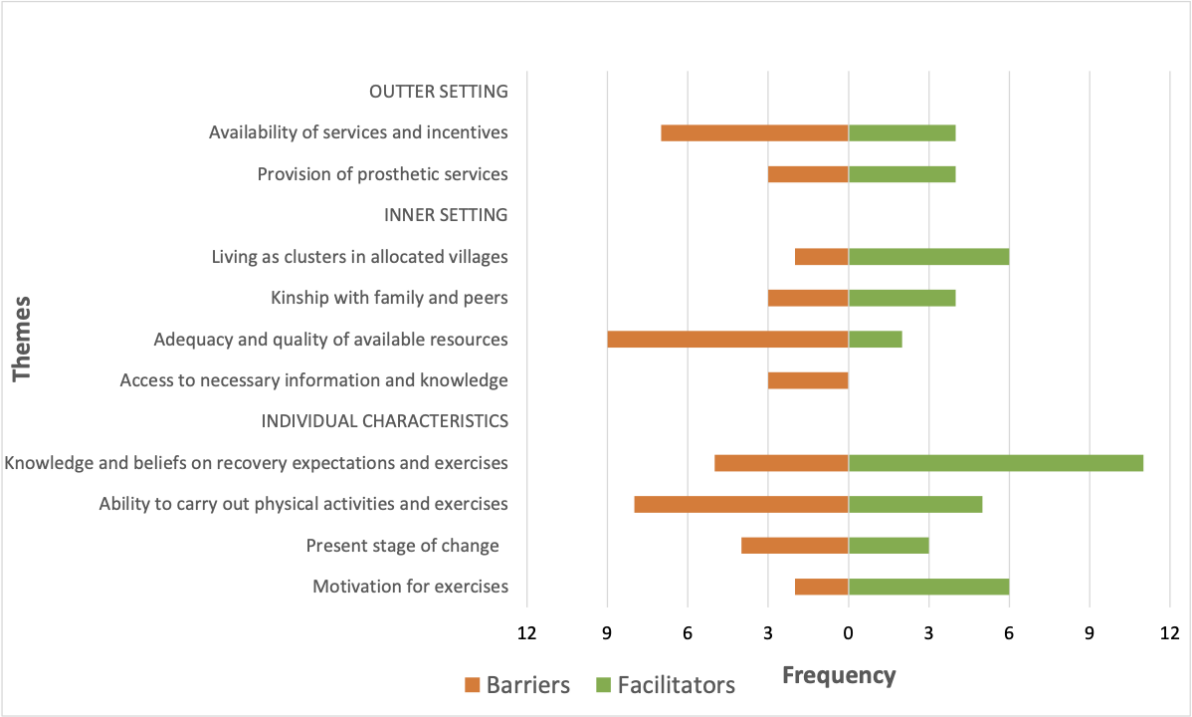
Importance of themes related to barriers and facilitators to physical activity participation as perceived by the veterans.

Barriers and facilitators were codified to 10 CFIR constructs within the major domains “outer setting,” “inner setting,” and “characteristics of individuals.” Table 5 provides a summary of emergent themes, their relationships with CFIR domains, and how they relate to barriers and facilitators to PA participation. Figure 1 shows the importance of themes as perceived by participants. Related participant quotes from the interviews are presented in Table 6.

**Table 5:** Perceived barriers and facilitators to physical activity participation and their associations with Consolidated Framework for Implementation Research domains.

CFIR^a^ domain and construct		Theme	Barrier	Facilitator
**Outer setting (broader external context in which the behavior or implementation occurs)**				
	External policies and incentives	Availability of services and incentives	Absence of community rehabilitation services	Financial support
	Patient needs and resources	Provision of prosthetic services	Unequal distribution of prosthetic services	Free of charge prosthetic services
**Inner setting (specific context within the organization or system where the behavior or implementation takes place)**				
	Structural characteristics	Living as clusters in allocated villages	Isolation from the wider society	Inclusive community environment
	Networks and communications	Kinship with family and peers	Family commitments	Family support Peer support Soldier societies
	Available resources	Adequacy and quality of available resources	Limited physical space at home Absence of exercise equipment Low-quality prosthetic legs	Calm environment in the village Adequate space in the village
	Access to information and knowledge	Access to necessary information and knowledge	Lack of access to knowledge and information on rehabilitation professional services	N/A^b^
**Individual characteristics (the personal attributes and characteristics of individuals performing the behavior or involved in the implementation)**				
	Knowledge and beliefs	Knowledge and beliefs on recovery expectations and exercises	Uncertainty of recovery expectations	Knowledge of the basic principles of exercise Preinjury active lifestyle
	Self-efficacy	Ability to carry out physical activities and exercises	Burden of chronic pain and persistent comorbidities Higher level of amputation	Active prosthetic use Age (middle-aged adult)
	Individual stage of change	Present stage of change	Present sedentary lifestyle	Current engagement in exercise
	Personal attributes	Motivation for exercises	Laziness	Motivation to be more active and independent Positive attitude toward exercise

^a^CFIR: Consolidated Framework for Implementation Research.

^b^N/A: not applicable.

**Table 6:** Representative participant quotes for themes related to the barriers and facilitators to physical activity participation.

Theme	Participant quotes
Availability of services and incentives	“Has anyone told that we’ve community rehabilitation centers to visit? ...we don’t have such rehabilitation services around our area, I don’t think we have such in the city even.” “No one has come to me and talked about doing exercises since I discharged from the hospital about 17 years ago.” “The thing is unlike for civil disabled people; we are given a monthly salary with an allowance. So, we don’t need to work hard on earning. You know then we have enough time to do some activities at home.”
Provision of prosthetic services	“Last year I was able to get a new prosthetic leg from a mobile social service program. I was directed to this by the president of our society. It is easy for me to work with this new one than the earlier one.”
Living as clusters in allocated villages	“As I think, there are about 24 villages in Sri Lanka that are reserved for veterans. So, all my neighbors are retired soldiers and the majority have the same disability like me. Normally we get together very often, and we can do exercises together.”
Kinship with family and peers	“I have to help my family members, especially my wife. She likes when I help to do household work. So, I should help with that work most of the time. She doesn’t care whether I’m doing exercises or not.” “My children don’t do their own work, so I have to help them as well, I have to bring them to school, tuition classes and stay there until they finish.”
Adequacy and quality of available resources	“There is no sufficient space at home to do exercises. If we have a separate room to continue exercises, it would be easier. I think none of us have that facility.” “Not having proper equipment is a barrier. I think to follow a physical rehabilitation program properly, we need suitable equipment.” “This prosthetic leg is the only means of mobility for me. But this is so heavy and already worn out. How can I do exercises with this? even it is difficult to walk with this.”
Access to necessary information and knowledge	“Although I want to do exercises, there is no one around to get proper information. But I do some exercises what I feel is good. Sometimes I do exercises to my leg using a sandbag as taught at the hospital.”
Knowledge and beliefs on recovery expectations and exercises	“Currently, I engage in many household activities like gardening and growing vegetables. I don’t feel it necessary to do any other special kind of exercise.” “...Yes, I engage in the normal day to day activities as much as I can. So, I think that is quite enough for the body as an exercise...” “We as soldiers had a good training on physical fitness and we know exercises better than a civil person. I mean before the injury we did exercises as part of our daily schedule in the Army.”
Ability to carry out physical activities and exercises	“The thing is I can’t use my body like I used to. Because my body, especially the back and the knee joints, start hurting when I start doing exercises. So, If I do exercises, I will not be able to do my normal routine the next day and sometimes I need to see a doctor after that to take medication for pain.” “For the sake of this prosthetic leg, I can walk when I want even as an exercise, otherwise I would just sit on a chair.”
Present stage of change	“I could manage to do the things and do exercises at this age but what will happen when I am old? I’m doing most of the activities in the paddy field because I have enough strength, because I’m still young.” “You know, most of us just eat and stay at one place and we are used to it, I don’t work as we did in the past, and even if I go somewhere, I just use my three-wheeler for that.”
Motivation for exercises	“...I don’t do exercises because I feel lazy to do...” “I don’t want to get my health worsen; I don’t like to be a burden to my wife and family. You know, usually soldiers like to keep their health in good condition and avoid troublesome diseases like diabetes.” “Although now we are disabled, we fought for the country for many years. At least I want to do my things independently and walk somewhere when I want, without wanting to trouble others.”

### Outer Setting

#### Availability of Formal Community-Based Rehabilitation Services

Participants lacked structured CBPR programs and community-based follow-up care from rehabilitation health providers, primarily due to the absence of formal community rehabilitation services like physiotherapy. They believed that having a CBPR program upon discharge from institutional care would have increased their PA participation.

#### Provision of Prosthetic Services

Prosthetic limbs were the only means of ambulation for the veterans included in this study, and they are needed to engage effectively in PA. Veterans are given free prosthetic legs by nongovernmental organizations to support their independent mobility. However, this service was not available all the time and was only available to a few of the participants. People who received this service had the opportunity to replace a worn-out prosthesis with a new one.

#### Financial Support

Continuous financial support from the government in the form of a monthly salary and disability allowance relieved participants of the burden of earning money for their households, enabling them to dedicate ample time to PA and exercise. However, this support led to sole reliance on the allowance, discouraging them from pursuing any occupational opportunities. This was connected to reduced participation in work-related PA and reduced motivation for PA, which has been described under the subsection “Individual Characteristics.”

### Inner Setting

#### Veterans’ Residence and Their Kinship

Veterans resided in designated villages allocated for army veterans, providing a peaceful environment with ample space for PA like walking and gardening. Living among peers with similar mental and physical states fostered an inclusive environment, where disabilities were not emphasized, encouraging frequent sharing of thoughts and experiences. Additionally, kinship with family and associated competing responsibilities hindered their engagement in PA.

#### Adequacy and Quality of Available Resources Required to Engage Effectively in PA

Participants did not have adequate space and equipment to engage in exercise and PA. They believed that exercise would not be effective without proper exercise equipment. The poor functionality of the prosthetic leg combined with skin wounds resulting from its incorrect fitting posed challenges for participating in PA, particularly walking activities.

#### Access to Information and Knowledge on Professional Services for Rehabilitation

Acquiring proper knowledge and training is crucial for successful and effective engagement in PA. However, participants expressed a lack of access to professionals or services to seek information and guidance on performing exercises at home.

### Individual Characteristics

#### Veterans’ Knowledge and Beliefs Regarding Recovery Expectations and Exercises

Participants expressed uncertainty about what to expect in terms of recovery upon discharge from inpatient care. They lacked an understanding of the importance of ongoing exercise engagement for their recovery, with some believing that exercises would not contribute further to their progress. Instead, they perceived activities, such as household chores, gardening, and walking to nearby shops or houses, as sufficient for maintaining a healthy life.

Owing to their active lifestyle before the injury (heavy physical training in the army and representing army sports teams), participants believed that they were familiar with the basic exercise principles. This helped them to engage in at least a few exercises at home even without proper guidance or follow-up.

#### Veterans’ Ability to Perform PA and Exercises

Veterans reported various health comorbidities, including back pain, knee pain, diabetes mellitus, and hypertension, which affected their ability to engage in PA. Veterans with transfemoral amputation perceived lower PA abilities compared to those with transtibial amputation, and they anticipated a worsening situation with age. In contrast, some veterans associated their current physical state positively with engaging in PA. One reason they mentioned was being an active prosthetic user, which made them independent in walking. As they joined the military service at 18-24 years of age and got injured at a young age, their relative age at the time of injury was seen as a facilitator to recovery.

#### Individual Motivation and Conflicting Priorities

Participants considered engaging in PA and exercise as an extra burden, requiring them to modify their usual lifestyle. Some expressed a lack of motivation for any form of PA, including walking for daily tasks. In contrast, for some participants, consistent engagement in PA was considered crucial among individuals with disabilities. It was seen as a lifelong requirement rather than a lifestyle choice to improve functional levels and reduce the risk of health issues, such as diabetes mellitus and heart disease. They expressed motivation to increase their activity levels and independence, aiming to avoid dependence on family members, including that related to the incidence of chronic health conditions.

### Expectations for a Future CBPR Program

**Figure 2:**
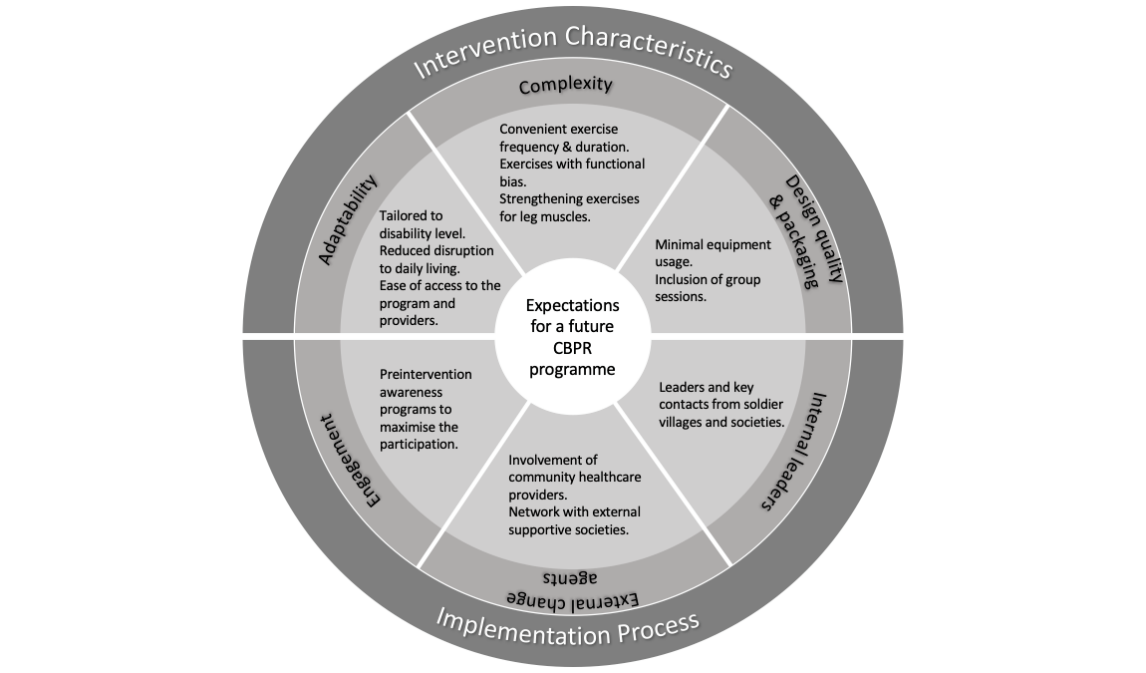
Perceived themes of expectations for a future community-based physical rehabilitation program and their associations with Consolidated Framework for Implementation Research domains. CBPR: community-based physical rehabilitation.

Twelve expectations for a future CBPR program emerged, and these were related to 6 constructs under 2 major domains of the CFIR model: “intervention characteristics” and “implementation process” (Figure 2). Most of the themes of expectations were related to “intervention characteristics.” Related participant quotes from the interviews are presented in Table 7.

**Table 7:** Representative participant quotes for themes of expectations for a future community-based physical rehabilitation program.

Theme	Participant quotes
Tailored to functional and disability levels	“Most importantly the exercise program should not be difficult to follow, especially the activities should match to us. You know, we cannot do activities similar to a normal individual who has both their legs.” “I won’t be able to perform difficult exercises, my back starts paining even after simple activities. So, I think I should follow a simple exercise program.”
Reduced disruption to daily living and ease of access	“I would do exercises at home. It is easy for me rather than travelling to a distance hospital without wasting time and money; travelling in public transport is a headache.” “Sometimes I do pushups at home before going to my shop. I can’t go anywhere else to do exercises, because I don’t have time, I should be there at the shop.”
Convenient exercise parameters and components	“Engaging in a daily exercise program would be very difficult, but 2-3 days per week would be fine.” “All I want is to engage properly in my farming activities, if the program can help me for that, it would be amazing.” “Before anything I want to walk more speedily, I’m getting slow and slow, it is embarrassing.” “I have seen my leg is getting thinner. If we get overweight, it will affect our legs as legs should bear the weight..., I think we should focus more on keeping our legs strong, especially the good leg.”
Use of equipment and space and involvement of peer groups	“I know some form of special exercises like pushups and squats do not need equipment. So, if these kinds of exercises are included in the program, it would be better.” “We live in this village together, so I think we can do exercises together in one common place, it would be more interesting”
Preintervention awareness programs	“It would be better if you can organize an awareness workshop for all of us before introducing the program. Otherwise, many of the veterans will miss this opportunity.”
Involvement of soldier societies and community health care providers	“There should be a person to contact when we have something to clarify when following the exercise program, actually, we will get many issues.” “Normally, if I need to talk to Army officials for any reason, all I do is contact president of our society and request to pass the message, I’m speaking about that kind of a process.” “All of us are members of the ‘Ranaviru Sansadaya’ and many of us are active members including me. I participate in almost all the events organized by this society. If you deliver the program through this society, it will surely become successful.”

### Intervention Characteristics

#### Tailored to Functional and Disability Levels

Participants expected the CBPR program to be tailored to their disability, with exercises matching their current functional levels. Exercises to prevent deterioration in existing health, notably back pain and knee pain, were a particular priority for participants with chronic comorbidities.

#### Reduced Disruption to Daily Living and Ease of Access

Veterans held a favorable perception toward CBPR, perceiving it as easily adaptable to their needs. They expressed a preference for engaging in rehabilitation programs either at home or within their local community, as opposed to attending outpatient clinics at hospitals. This preference was associated with perceived benefits, such as reduced travel burden, lower associated costs, and minimized disruptions to their daily lives.

#### Convenient Exercise Parameters and Components

Veterans generally suggested a program with 2 to 3 sessions per week, lasting 20-30 minutes each. They favored simple functionally oriented exercises that could be easily incorporated into daily activities, with a preference for specific exercises, such as leg muscle strengthening.

#### Use of Equipment and Space and Involvement of Peer Groups

Veterans preferred using exercise equipment only when necessary, considering constraints like the lack of equipment at home and financial limitations. They showed interest in using community spaces, such as playgrounds and meeting halls, for group rehabilitation sessions when home space was insufficient. Group participation was favored for the opportunity to learn from one another during the program.

### Process of Implementation

#### Preintervention Awareness Program

Participants stressed the importance of an awareness program led by experts in the field to precede the implementation of a future CBPR program, with the aim of ensuring maximum engagement of veterans in the CBPR program.

#### Involvement of Soldier Societies and Community Health Care Providers

Veterans highlighted the necessity of key contact from both veterans and rehabilitation providers for each village. This is to communicate the necessary information smoothly and get advice when necessary.

The veterans were members of formal societies like “Ranaviru Sansadaya,” which are associated with enabling participants to stay united as one group of army veterans and connecting them with external organizations to receive help. They anticipated that delivering the program through these societies would help initiate and continue the program successfully.

### Integration

**Figure 3:**
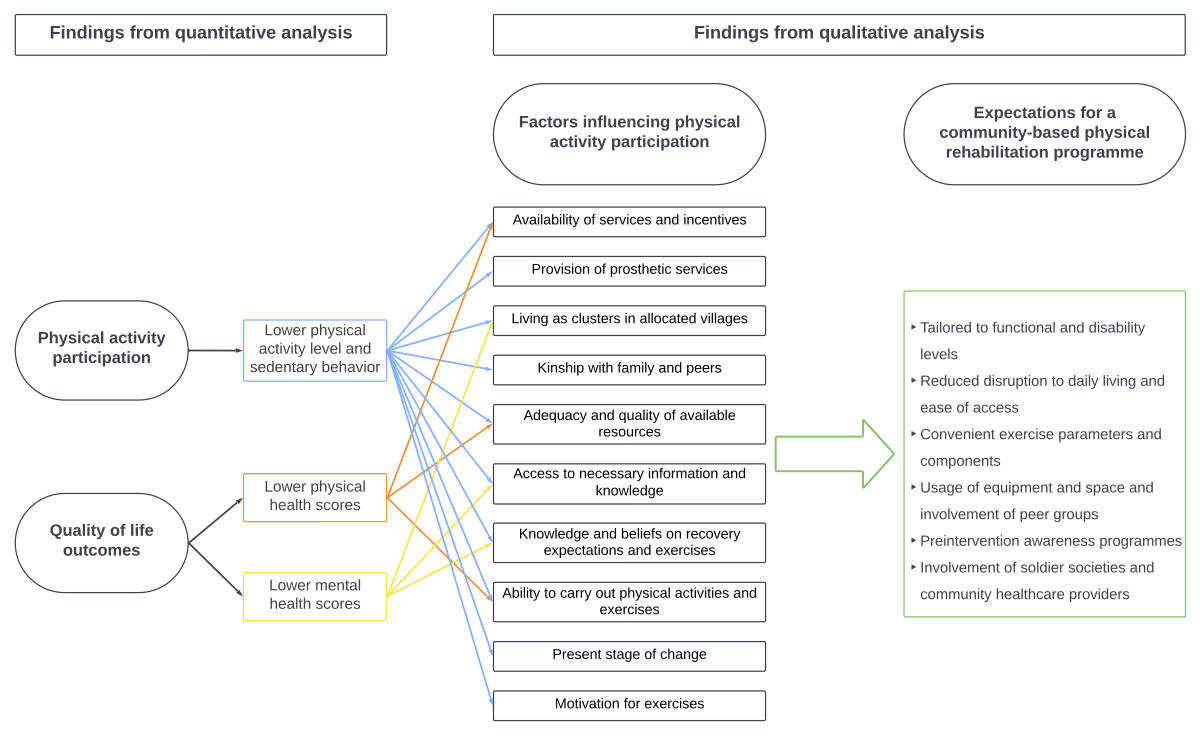
Joint display of quantitative and qualitative findings.

Figure 3 presents the joint display of quantitative and qualitative findings. Themes of the barriers and facilitators to PA were identified as factors influencing PA participation among veterans, which were associated with lower PA levels and sedentary behavior observed among the majority of the veterans. Additionally, some of these themes were linked to lower QoL outcomes in both physical and mental health domains. Of the themes that were linked to both QoL outcomes and PA participation, themes, such as availability of services and incentives, adequacy and quality of available resources, knowledge and beliefs on recovery expectations and exercises, and ability to carry out PA and exercises, emerged with high frequencies (Figure 1). Expectations for a future CBPR program, which was identified as a potential solution to improve QoL and PA participation by addressing influential factors, are also presented in Figure 3.

## Discussion

### Principal Findings

Limited availability of and access to community-based rehabilitation and prosthetic services for survivors of LLA have resulted in poor levels of physical mobility that affect QoL both physically and mentally, including the ability to work, compared with able-bodied members of the society in Sri Lanka. The strongest barriers to PA include low-quality prosthetics and a growing burden of comorbidities, leading to fear and discomfort during PA. A preinjury active lifestyle and a positive attitude toward exercise, especially with family and peer support, were identified as crucial for sustained mobility and long-term rehabilitation. Expectations for a CBPR program included community-based activities tailored to individual disability levels, which are supported by peers and health care providers and are feasible for completion at home.

### QoL Outcomes

This study revealed lower QoL outcomes among veterans compared with the findings in a previous study conducted over 20 years ago on the same population [25]. This suggests a decline in QoL over time, possibly attributed to reduced PA participation and rising comorbidities associated with a sedentary lifestyle and poorly managed pain and discomfort [49]. Veterans perceived a decline in their ability to engage in PA and associated it with aging and comorbidities, such as back pain, knee joint pain, hypertension, and diabetes. Consistent with the findings of this study, lower QoL outcomes have been observed among individuals with LLA than among the general population internationally [4,7,50-53].

### PA Participation and Influential Factors

Usually, before injury, soldiers have higher levels of PA for their age range compared with nonservice community members. Despite this anticipated higher baseline, survivors of LLA had limited physical function, and their injury was associated with poor functional activity and mental well-being. The survey findings indicated that veterans primarily engaged in moderate-intensity PA, such as gardening, with minimal participation in vigorous-intensity PA, such as sports. Interviews further clarified that veterans perceived activities like household chores, gardening, and walking to nearby shops or houses as sufficient for maintaining a healthy life. However, they failed to meet the recommended levels of PA for an average adult. Their scores were lower compared to scores in similar studies conducted in Australia and the United States, where PA and medium- to long-term community-based rehabilitation programs, including sports activities led by veterans and peer groups, are well established [40,54,55].

Although kinship with peer veterans having similar disabilities was perceived as a facilitator for engaging in PA, living in isolation from the wider society may have contributed to the normalization of their sedentary behavior, which may further be aggravated by the lack of knowledge of recovery expectations and the prevention and management of potential health comorbidities.

### Expectations for a Future CBPR Program

Important aspects regarding expectations for a future CBPR program perceived by participants of this study could be described in the following 3 key areas: individualization; function-based exercises; and involvement of key resource persons like peers with LLA, amputee societies, and community health care providers. Tailoring intervention components to individual baseline parameters, such as age, disability level, and home environment, is considered essential for participant engagement. Functional exercises are performed with the purpose of enhancing basic everyday motor performance (eg, walking, stair climbing, or sitting and standing up from a chair) and are based on the exercise training principle of specificity [56]. Emphasizing a high functional bias in intervention components reduces the reliance on specialized exercise equipment and allows participants to relate the program to their normal daily activities more easily. For example, use of a graded community walking program and step-ups onto a platform instead of treadmill walking and using bodyweight exercises, such as squats, lunges, and push-ups, to improve muscular strength. The involvement of peers with similar disabilities, amputee societies, and community health care professionals is important in all stages of a community-based rehabilitation program (from design to implementation and follow-up). Similarly, a study highlighted that rehabilitation professionals perceived the involvement of committed and enthusiastic individuals as necessary for the successful implementation and ongoing promotion of PA in the rehabilitation of people with disabilities [57].

### Strengths and Limitations

Our study employed both quantitative and qualitative data to investigate PA levels and explore the rationale behind the results and participants’ perspectives on potential solutions. The use of theoretical frameworks and adherence to recommended guidelines strengthened our research. However, the generalizability of the findings is limited to male veterans with war-related traumatic unilateral LLA in the community. Nonetheless, our findings shed light on the experiences of a specific disadvantaged group of individuals living in a low-resource setting. Although self-report measures may introduce bias, we mitigated this by using an adequate sample size and a matched control group.

### Conclusions

The decline in overall well-being among veterans with LLA in Sri Lanka over time underscores their unmet rehabilitation needs and reveals the long-term impacts of living with LLA in the absence of physical rehabilitation for a young group of veterans. The majority of participants with LLA exhibited insufficient levels of PA owing to barriers, including the absence of community rehabilitation services, limited resources, and a growing burden of comorbidities, such as chronic pain and psychological distress. A future CBPR intervention that is individualized to meet the needs of survivors, with a focus on functionality-biased exercises, and is led by and delivered with peer societies and community health care providers is considered fundamental for successful implementation and adoption. Among the participants, high receptivity in the implementation climate, peer support, a preinjury active lifestyle, and motivation and positive attitudes toward exercise emerged as strong indicators of engagement in a future CBPR program.

### Implications for Rehabilitation Practice and Policy

Improving PA participation to recommended levels and enhancing QoL in both physical and psychosocial aspects should be prioritized in the design and implementation of CBPR interventions targeted at individuals with LLA in similar contexts. As the studied population lived with amputation for more than 10 years and the majority had a low to sedentary level of PA, behavior change mechanisms should be incorporated in the intervention components aimed at improving PA participation [58,59]. For effectively addressing the identified challenges, it is required to ensure fair access to community-based rehabilitation services, provide veterans and their families with essential knowledge, and foster support networks through policy-level changes.

### Recommendations for Future Research

Future studies should aim to identify the determinants of low QoL and PA participation observed among veterans in this study. Additionally, it is crucial to establish specific PA and exercise parameters effective for improving health outcomes within this LLA subgroup, which need to be considered in a future CBPR program. To enhance the feasibility of future CBPR interventions, inclusive representation of various stakeholders, including health care providers, social workers, and family members, through future qualitative studies is recommended. Furthermore, the feasibility and cost-effectiveness of such CBPR interventions in low-resource settings should be assessed in high-quality randomized controlled trials. As this study was conducted in military community settlements where the majority of veterans with LLA live, the living environment and associated factors like social support and access to rehabilitation services would be different from those of civilians with LLA. In addition, the causes of amputation (traumatic vs vascular), preamputation job roles, and PA levels between military veterans and civilians are generally different. Therefore, repeating the examinations conducted in this study in the civilian population with LLA is crucial for effectively adapting the proposed CBPR program to this population.

## Appendix

Multimedia Appendix 1 Interview guide.

Multimedia Appendix 2 Comparison of quality of life and physical activity participation between veterans with transfemoral amputation and those with transtibial amputation.

Multimedia Appendix 3 Participation in specific forms of physical activities in group 1 (veterans with lower limb amputation).

## Abbreviations

CBPR: community-based physical rehabilitation
CFIR: Consolidated Framework for Implementation Research
GN: Grama Niladhari
IPAQ: International Physical Activity Questionnaire
LLA: lower limb amputation
MCS: mental component summary
MET: metabolic equivalent of task
PA: physical activity
PCS: physical component summary
QoL: quality of life
SF-36: Short-Form Health Survey-36
