# Supplementary material for: Quality of Life, Physical Activity Participation, and Perceptions of Physical Rehabilitation Among Community-Reintegrated Veterans With Lower Limb Amputation in Sri Lanka: Convergent Parallel Mixed Methods Study
Source: JMIR Rehabil Assist Technol. 2024 Jun 13;11:e52811. doi: 10.2196/52811 (PMC11211708; doi:10.2196/52811)
Supplement: Multimedia Appendix 1 [file rehab_v11i1e52811_app1.docx]

**Multimedia Appendix 1.** Interview guide.

Perceptions of barriers and facilitators to physical activity participation and expectations for a future community-based physical rehabilitation programme among community-reintegrated veterans with lower limb amputation in Sri Lanka.

**Interview Guide**

1. **Immediate postamputation rehabilitation and expectations of recovery**
2. What difference has the amputation made to your life?
3. Tell me about the rehabilitation you received after your amputation. What kinds of exercise did you do as part of your rehabilitation?
4. What are your expectations of recovery?
5. What are your expectations of the time it would take to recover?
6. **Exercise and physical activity participation**
7. Describe your typical day, including the activities and exercises you usually do.

*Probes*

- What kinds of activities do you do which keep you active during a typical day? (e.g. walking and gardening)
- Please describe what do you know about exercises and exercise programs.
- Do you do any specific exercises to improve your strength or fitness? If so, tell me about the exercises. How often do you do them? In what setting? With whom?
- How has your activity level changed since you had an amputation?
- What physical activity and/or exercises did you do before your amputation?

1. **Barriers and Facilitators to physical activity participation**
2. What has helped you to be physically active?
3. What has helped you participate in structured exercise?
4. What are the barriers that prevent you from being physically active?
5. What are the barriers that prevent you from participating in structured exercise?

*Probes*

- Do you think physical activity is important? Explain why or why not.
- How do you feel about engaging in physical activities and/or exercises?
- How easy or difficult is it for you to do physical activities?
- What makes you confident in doing physical activity or lack confidence?
- How confident are you to do specific exercises (e.g. strengthening exercises)?
- What do you think will happen if you participate in more physical activity or exercise?
- Tell me about any barriers in your home or community that make it difficult to be physically active.
- Tell me about any supports or resources you have that help you stay active.

1. **Expectations for a future community-based physical rehabilitation programme**
2. What are your suggestions on starting and continuing a physical rehabilitation programme at your home/community?

*Probes*

- What kinds of activities or exercise do you like to have in the programme?
- What is the duration of the programme you think is feasible to you?
- How many times per day you think you could engage in such a programe?
- What kind of exercise equipment you like to use?
- What kind of support or resources do you need to participate in the rehabilitation programme?
- How would you prefer to get information about the programme? From whom?
- Would you prefer a group versus individual program? Explain.
- How do you think the adherence for such a programme could be improved?

1. Is there anything else you that we have not discussed that you feel is important or that you would like to talk about more?
