# Supplementary material for: Quality of Life, Physical Activity Participation, and Perceptions of Physical Rehabilitation Among Community-Reintegrated Veterans With Lower Limb Amputation in Sri Lanka: Convergent Parallel Mixed Methods Study
Source: JMIR Rehabil Assist Technol. 2024 Jun 13;11:e52811. doi: 10.2196/52811 (PMC11211708; doi:10.2196/52811)
Supplement: Multimedia Appendix 2 [file rehab_v11i1e52811_app2.docx]

**Multimedia Appendix 2.** Comparison of quality of life and physical activity participation between veterans with transfemoral amputation and those with transtibial amputation.

| Quality of life / Physical activity participation | Transfemoral (n = 5) | Transtibial (n = 74) | *P* value^a^ |
| --- | --- | --- | --- |
| Quality of life Median (IQR) | | |  |
| Physical health  Physical functioning (PF)  Role limitation due to physical problems (RLPH)  Bodily pain (BP)  General health (GH)  Physical health component (PCS) | 50 (60-35)  50 (75-50)  67.5 (77.5-55)  35 (40-25)  53.1 (56.9-43.1) | 60 (75-45)  50 (75-25)  67.5 (77.5-55)  45 (56.3-40)^c^  55.3 (66.4-44.8) | .088  .263  .540  .009  .533 |
| Mental health  Role limitation due to emotional problem (RLEP)  Social functioning (SF)  Vitality (VT)  Emotional well-being (EW)  Mental health component (MCS) | 100 (100-66.7)  62.5 (75-62.5)  60 (65-50)  48 (64-40)  63.8 (71.6-61.8) | 66.7 (100-33.3)  75 (87.5-62.5)  60 (70-50)  56 (60-48)  59.3 (71.3-48.6) | .074  .439  .936  .289  .354 |
| Physical activity participation | | |  |
| Total physical activity level  (Median MET^b^-minute/week (IQR)) | 480.4 (378-598.5) | 1968.5 (560.3-3646.9) | .079 |
| Physical activity domains  (Median MET-minute/week (IQR))  Work  Transport  Domestic and garden  Leisure | 0.0 (0.0-0.0)  0.0 (0.0-0.0)  472.5 (283.5-535.5)  0.0 (0.0-0.0) | 0.0 (0.0-686.4)  0.0 (0.0-207.9)  803.3 (411.1-2480.6)  0.0 (0.0-155.93) | .416  .134  .053  .055 |
| Physical activity intensity  (Median MET-minute/week (IQR))  Total walking  Total moderate intensity activity  Total vigorous intensity activity | 0.0 (0.0-0.0)  480.3 (378-708.8)  0.0 (0.0-0.0) | 207.9 (0.0-688.7)^c^  1229.1 (515.8-3144.1)  0.0 (0.0-252) | .01  .059  .520 |
| Physical activity behaviour  (n (%))  Sedentary  Low  Sufficiently active | 4 (80)  0 (0)  1 (20) | 30 (40.5)  25 (33.8)  19 (25.7) |  |

^a^Statistical significance was assessed using the Mann–Whitney U test for comparison between transfemoral and transtibial amputations.

^b^MET: metabolic equivalent of task

^c^Statistical significance at *P*<.05.
