# Supplementary figures and images for: Quality of Life, Physical Activity Participation, and Perceptions of Physical Rehabilitation Among Community-Reintegrated Veterans With Lower Limb Amputation in Sri Lanka: Convergent Parallel Mixed Methods Study
Source: JMIR Rehabil Assist Technol. 2024 Jun 13;11:e52811. doi: 10.2196/52811 (PMC11211708; doi:10.2196/52811)

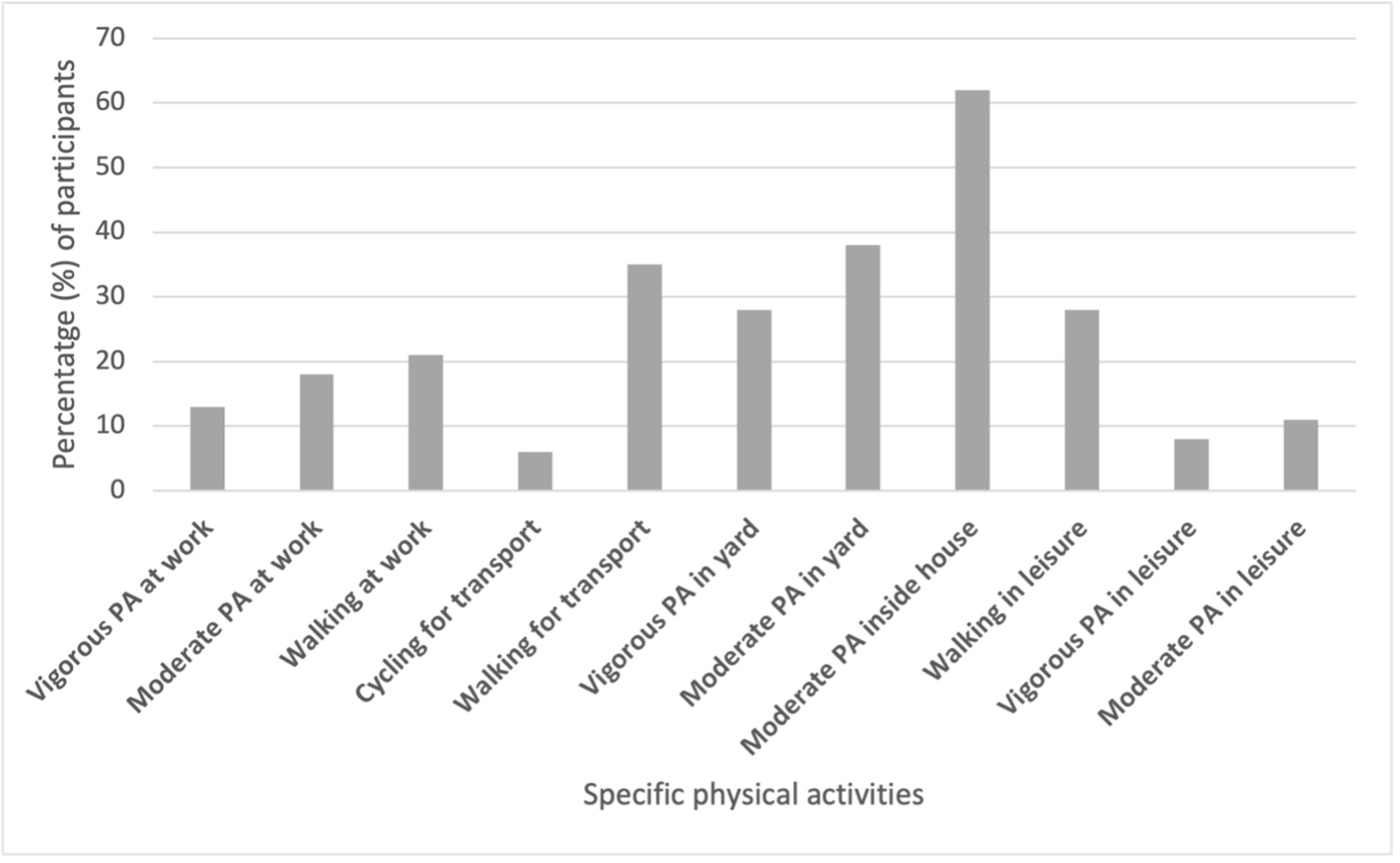

Supplement: Multimedia Appendix 3 [file rehab_v11i1e52811_app3.png]
